# Supplementary material for: rTMS Reduces Craving and Alcohol Use in Patients with Alcohol Use Disorder: Results of a Randomized, Sham-Controlled Clinical Trial
Source: J Clin Med. 2022 Feb 11;11(4):951. doi: 10.3390/jcm11040951 (PMC8878126; doi:10.3390/jcm11040951)
Supplement: Supplementary file 1 [file jcm-11-00951-s001.zip › jcm-1545087-supplementary.pdf]

Supplementary Materials:

Table S1: ANOVA tests for three outcome variables of craving

	Wilks' Λ	sig	F	df
VAS	0.490	0.001	6.502	4, 25
OCDS-5	0.729	0.084	2.323	4, 25
AUQ	0.604	0.011	4.095	4. 25

Table S2: MANOVA tests on different time points for craving variables

	Wilks' Λ	sig	F	df
10 days	0.901	0.430	0.952	3, 26
1 month	0.649	0.010	4.681	3, 26
3 months	0.492	0.000	8.949	3, 26
12 months	0.790	0.101	2.297	3, 26

Table S3 (a) Craving MANOVA without participants with PTSS at baseline

	Wilks' Λ	sig	F	df
	0.174	0,019	3.943	12, 10

Table S3 (b) ANOVA tests for three outcome variables of craving,without participants with PTSS at baseline

	Wilks' Λ	sig	F	df
VAS	0,399	0.002	6.788	4, 18
OCDS-5	0.665	0.102	2.265	4, 18
AUQ	0.490	0.009	4.676	4, 18

Table S4: Overview alcohol outcome measurements after one year of follow-up

	Total (N=30)		Real rTMS + TAU (n=14)		Sham + TAU (n=16)		F	p
	M	SD	M	SD	M	SD		
% Abstinence at endpoint	0.06		0,14		0,00		14.37	0.126
Alcohol use total in mg	4604	3500	3161	2716	5866	3694	1.349	0.032
Alcohol use per day in mg	45.50	34,60	31.27	26,80	57.94	36.59	1.42	0.03
Time to relapse	181.0	154,9	256.4	146.8	115.1	133.2	0.458	0.010
Total number of abstinent days	48.50	29,11	7007	23.44	29.63	18.65	0.004	0.000
Total number of HDD days	37.03	25,82	25.36	20.18	47.25	26.41	1.478	0.018
